# Association between HLA-B Alleles and Carbamazepine-Induced Maculopapular Exanthema and Severe Cutaneous Reactions in Thai Patients

**DOI:** 10.1155/2018/2780272

**Published:** 2018-01-10

**Authors:** Chonlaphat Sukasem, Chonlawat Chaichan, Thapanat Nakkrut, Patompong Satapornpong, Kanoot Jaruthamsophon, Thawinee Jantararoungtong, Napatrupron Koomdee, Suthida Sririttha, Sadeep Medhasi, Sarawut Oo-Puthinan, Ticha Rerkpattanapipat, Jettanong Klaewsongkram, Pawinee Rerknimitr, Papapit Tuchinda, Leena Chularojanamontri, Napatra Tovanabutra, Apichaya Puangpetch, Wichai Aekplakorn

**Affiliations:** ^1^Division of Pharmacogenomics and Personalized Medicine, Department of Pathology, Faculty of Medicine Ramathibodi Hospital, Mahidol University, Bangkok, Thailand; ^2^Laboratory for Pharmacogenomics, Somdech Phra Debaratana Medical Center (SDMC), Ramathibodi Hospital, Bangkok, Thailand; ^3^Ramathibodi Multidisciplinary Epilepsy Center, Faculty of Medicine, Ramathibodi Hospital, Mahidol University, Bangkok, Thailand; ^4^The Thai Severe Cutaneous Adverse Drug Reaction (THAI-SCAR) Research Group, Bangkok, Thailand; ^5^Department of Pharmacy Practice, Faculty of Pharmaceutical Sciences, Naresuan University, Phitsanulok, Thailand; ^6^Department of Pharmacy, Prasat Neurological Institute, Bangkok, Thailand; ^7^Department of Pathology, Faculty of Medicine, Prince of Songkla University, Songkla, Thailand; ^8^Department of Clinical Pharmacy Practice, Faculty of Pharmacy, Mahidol University, Bangkok, Thailand; ^9^School of Medicine, Mae Fah Luang University, Chiang Rai, Thailand; ^10^Division of Allergy Immunology and Rheumatology, Department of Medicine, Faculty of Medicine, Ramathibodi Hospital, Mahidol University, Bangkok, Thailand; ^11^Division of Allergy and Clinical Immunology, Skin and Allergy Research Unit, Department of Medicine, Faculty of Medicine, Chulalongkorn University, Bangkok, Thailand; ^12^Division of Dermatology, Skin and Allergy Research Unit, Department of Medicine, Faculty of Medicine, Chulalongkorn University, Bangkok, Thailand; ^13^Department of Dermatology, Faculty of Medicine Siriraj Hospital, Mahidol University, Bangkok, Thailand; ^14^Dermatological Division, Department of Internal Medicine, Chiang Mai University, Chiang Mai, Thailand; ^15^Department of Community Medicine, Ramathibodi Hospital, Mahidol University, Bangkok, Thailand

## Abstract

The *HLA-B*∗*15:02* allele has been reported to have a strong association with carbamazepine-induced Stevens-Johnson syndrome/toxic epidermal necrolysis (SJS/TEN) in Thai patients. The *HLA-B* alleles associated with carbamazepine-induced maculopapular exanthema (MPE) and the drug reaction with eosinophilia and systemic symptoms (DRESS) among the Thai population have never been reported. The aim of the present study was to carry out an analysis of the involvement of *HLA-B* alleles in carbamazepine-induced cutaneous adverse drug reactions (cADRs) in the Thai population. A case-control study was performed by genotyping the *HLA-B* alleles of Thai carbamazepine-induced hypersensitivity reaction patients (17 MPE, 16 SJS/TEN, and 5 DRESS) and 271 carbamazepine-tolerant controls. We also recruited 470 healthy Thai candidate subjects who had not taken carbamazepine. *HLA-B*∗*15:02* showed a significant association with carbamazepine-induced MPE (*P* = 0.0022, odds ratio (OR) (95% confidence interval [CI]) = 7.27 (2.04–25.97)) and carbamazepine-induced SJS/TEN (*P* = 4.46 × 10^−13^; OR (95% CI) = 70.91(19.67–255.65)) when compared with carbamazepine-tolerant controls. Carbamazepine-induced SJS/TEN also showed an association with *HLA-B*∗*15:21* allele (*P* = 0.013; OR (95% CI) = 9.54 (1.61–56.57)) when compared with carbamazepine-tolerant controls. *HLA-B*∗*58:01* allele was significantly related to carbamazepine-induced MPE (*P* = 0.007; OR (95% CI) = 4.73 (1.53–14.66)) and DRESS (*P* = 0.0315; OR (95% CI) = 7.55 (1.20–47.58)) when compared with carbamazepine-tolerant controls. These alleles may serve as markers to predict carbamazepine-induced cADRs in the Thai population.

## 1. Introduction

Hypersensitivity reactions such as maculopapular exanthema (MPE), Stevens-Johnson syndrome (SJS), toxic epidermal necrolysis (TEN), and drug reaction with eosinophilia and systemic symptoms (DRESS) are common with carbamazepine therapy [1]. MPE is characterized by a diffuse cutaneous erythema which can evolve into severe forms, presenting as vesicles and papules [2]. SJS and TEN, being severe and fatal hypersensitivity reactions, are characterized by epidermal necrosis and skin detachment [3]. The percentage of body surface involvement in SJS is <10%, SJS/TEN overlap is 10%–30%, and TEN is >30% [4]. DRESS includes serious maculopapular eruptions, fever, pharyngitis, eosinophilia, and systemic symptoms with an estimated mortality rate of up to 10% [5–7]. SJS and TEN are bullous reactions, whereas MPE and DRESS are nonbullous reactions [8].

Investigators have found strong phenotype- and ethnicity-specific associations between carbamazepine-induced hypersensitivity reactions and human leukocyte antigen (*HLA*) genes [9–11]. In 2004, Chung et al. reported a very strong association between carbamazepine-induced SJS and *HLA-B*∗*15:02* allele in Han Chinese patients [12]. This study did not discuss *HLA* association with other cADRs associated with carbamazepine. The Food and Drug Administration (FDA) of the USA and the Clinical Pharmacogenetics Implementation Consortium (CPIC) have recommended screening for the *HLA-B*∗*15:02* allele prior to initiating treatment with carbamazepine in patients with Asian ancestry [13, 14]. The association of the *HLA-B*∗*15:02* allele with carbamazepine-induced SJS and TEN was reported in a systematic review and meta-analysis of the relationship between the *HLA-B*∗*15:02* allele and carbamazepine-induced SJS and TEN among Han Chinese, Thai, and Malaysian populations [15]. Grover and Kukreti, in a meta-analysis study exploring the relationship between *HLA* alleles and carbamazepine-induced cutaneous adverse drug reactions (cADRs) among Asian patients treated with carbamazepine, showed an association of cases of carbamazepine-induced SJS and TEN with *HLA-B*∗*15:02* and *HLA-B*∗*15:11* alleles [16]. The authors also showed an association between cases of MPE, DRESS, and SJS/TEN caused by carbamazepine and the *HLA-A*∗*31:01* allele. The *HLA-A*∗*31:01* allele was reported to be associated with carbamazepine-induced hypersensitivity reactions among the subjects of European descent [17]. The *HLA-A*∗*31:01* allele was significantly associated and was a distinct genetic predictor of carbamazepine-induced DRESS but not for carbamazepine-induced SJS/TEN in Chinese and Europeans [18]. Patients with carbamazepine-induced MPE/DRESS showed an association with the *HLA-A*∗*31:01* and *HLA-B*∗*51:01* alleles in a study performed in Han Chinese patients [19].

The association between the occurrence of carbamazepine-induced cADRs and the *HLA* allele among the Thai population has been reported previously in only one study. In a case-control study in a Thai population, Tassaneeyakul et al. found a strong association between the presence of the *HLA-B*∗*15:02* allele and SJS/TEN induced by carbamazepine [20]. More recently, a Thai patient with carbamazepine-induced SJS did not show the presence of the *HLA-B*∗*15:02* allele but showed the presence of the *HLA-B*∗*15:21* allele [21]. There is no published data of genetic association of carbamazepine-induced MPE and DRESS within the Thai population. In the present study, we sought to investigate the *HLA-B* allele-phenotype correlations in carbamazepine-induced MPE, DRESS, and SJS/TEN in Thai subjects.

## 2. Materials and Methods

### 2.1. Subjects and Characteristics

This study was carried out as a retrospective and prospective case-control study. From 2011 to 2016, patients with carbamazepine-induced cADRs were retrospectively and prospectively enrolled from the Faculty of Medicine Ramathibodi Hospital, Mahidol University, the Faculty of Medicine, Chulalongkorn University, Prasart Neurological Institute, and the Thai Severe Cutaneous Adverse Drug Reaction (THAI-SCAR) research group, Bangkok, Thailand. Among them, 38 patients with carbamazepine-induced cADRs were categorized into MPE (17 cases), SJS/TEN (16 cases), and DRESS (5 cases). Meanwhile, patients who had been taking carbamazepine for more than 6 months without evidence of cutaneous adverse effects were recruited as carbamazepine-tolerant controls (*n* = 271). In addition, 470 healthy Thai subjects were recruited who were not taking carbamazepine. The study was approved by the Ethical Review Committee on Research Involving Human Subjects, Faculty of Medicine, Ramathibodi Hospital, Mahidol University.

### 2.2. Diagnosis of Carbamazepine-Induced Cutaneous Adverse Drug Reactions

Hypersensitivity reactions were classified according to the criteria of the RegiSCAR study, and a dermatologist and an allergist confirmed the diagnoses on the basis of the photographs, pathological slides, clinical morphology of the skin damage, and medical records [22].

MPE was defined as cutaneous fine pink macules and papules and lesions without mucosal or systemic symptoms [23]. SJS/TEN cases were defined according to the detached body surface area as SJS (3–10%) and SJS/TEN overlap (10–30%) with or without associated systemic symptoms but not fulfilling the criteria of DRESS [22]. DRESS was defined as follows: presence of fever, maculopapular rash with internal organ involvement, and hematologic abnormalities [24].

### 2.3. DNA Isolation and *HLA-B* Typing

DNA extraction (MagNA Pure Compact nucleic acid purification kit, Roche Diagnostics Ltd., USA) was performed based on magnetic bead technology. DNA was aliquoted and stored at −20°C before *HLA* typing. *HLA-B* alleles were analyzed by the polymerase chain reaction-sequence-specific oligonucleotide probe (PCR-SSOP) assay and Luminex™ Multiplex Technology with well-established protocols [22]. In brief, PCR products were hybridized against a panel of oligonucleotide probes coated on polystyrene microspheres that have sequences complementary to stretches of polymorphic sequence within the target *HLA-B* alleles. The amplicon-probe complex was visualized using a colorimetric reaction and fluorescence detection technology. Data analysis for the *HLA-B* assays was performed with HLA fusionTM2.0 software.

### 2.4. Statistical Analysis

Statistical analysis was performed with SPSS version 18.0 (SPSS Inc., Chicago, IL, USA). Allele case-control comparisons were analyzed by Fisher's exact test. A two-sided *P* value < 0.05 was considered to be statistically significant.

## 3. Results

### 3.1. Subjects

Table 1 summarizes the clinical manifestations and demographic variables of the 38 cases and 271 carbamazepine-tolerant controls. Most cases received carbamazepine to treat epilepsy (29 cases), except for 9 patients who received carbamazepine to treat trigeminal neuralgia (5 cases), neuropathic pain (2 cases), bipolar disorder (1 case), and paroxysmal kinesigenic and nonkinesigenic dyskinesia (1 case). The mean treatment dose of carbamazepine in the carbamazepine-induced cADR patients was 325 ± 75 mg/day (mean ± standard deviation). There was no significant differences between the case and tolerant group in treatment dose of carbamazepine. The mean duration for the onset of cADR was 16 ± 7 days (mean ± standard deviation).

### 3.2. Association of *HLA-B* Alleles with Carbamazepine-Induced cADRs

Of the 38 patients who had carbamazepine-induced cADRs, 17 (44.74%) were found to carry the *HLA-B*∗*15:02* allele. The *HLA-B*∗*15:02* allele was observed in 4.06% (11/271) of carbamazepine-tolerant controls and 15.11% (71/470) of the general Thai population (Table 2). Our analysis of all subjects with cADRs and clinical control subjects showed a significant allelic association with *HLA-B*∗*15:02* (*P* = 7.35 × 10^−12^), generating an odds ratio (OR) of 19.13 (95% confidence interval [CI], 7.94–46.09). A comparison of all 38 carbamazepine-induced cADR subjects with 470 general Thai subjects produced an OR of 4.55 (95% CI, 2.29–9.05, *P* = 3.44 × 10^−6^). Two patients with carbamazepine-induced cADRs carried *HLA-B*∗*15:21*, while the other *HLA-B* serotypes 75 were not detected in this study.

### 3.3. Association between *HLA-B* Alleles and Various Types of Carbamazepine-Induced cADRs

We analyzed the *HLA-B* association between 17 patients with carbamazepine-induced MPE and 271 carbamazepine-tolerant controls. We found two *HLA-B* alleles, *HLA-B*∗*15:02* and *HLA-B*∗*58:01*, as significant in the carbamazepine-induced MPE (Table 3). The *HLA-B*∗*15:02* allele was observed in 23.53% (4/17) of patients with carbamazepine-induced MPE, but only in 4.06% (11/271) of the carbamazepine-tolerant controls, giving a significant association with carbamazepine-induced MPE (*P* = 0.002; OR (95% CI) = 7.27 (2.04–25.97)). The *HLA-B*∗*58:01* allele appeared in 29.41% (5/17) of patients with carbamazepine-induced MPE, which was more frequent than in carbamazepine-tolerant controls (8.12%, 22/271; *P* = 0.007; OR (95% CI) = 4.73 (1.53–14.66)). In the included general population, the carrier rates of *HLA-B*∗*15:02* and *HLA-B*∗*5801* were 12.34% (58/470) and 12.13% (57/470), respectively. Comparing the difference of the *HLA-B*∗*58:01* allele frequencies between the 17 patients with carbamazepine-induced MPE and 470 general subjects, *HLA-B*∗*58:01* showed the significant association with carbamazepine-induced MPE (*P* = 0.045; OR (95% CI) = 3.02 (1.03–8.88)). As for the carbamazepine-induced SJS/TEN, the *HLA-B*∗*15:02* and *HLA-B*∗*15:21* alleles were most significantly detected (Table 4). 75% (12/16) of carbamazepine-induced SJS/TEN patients carried *HLA-B*∗*15:02*, which was more frequent than in carbamazepine-tolerant controls (4.1%, 11/271; *P* = 4.46 × 10^−13^; OR (95% CI) = 70.91 (19.67–255.65)). The *HLA-B*∗*15:02* allele was present in 15.11% (71/470) of the general population and when we compared the difference of *HLA-B*∗*15:02* frequency between carbamazepine-induced SJS/TEN patients and the general population, *HLA-B*∗*15:02* showed a significant association with carbamazepine-induced SJS/TEN (*P* = 6.9 × 10^−8^; OR (95% CI) = 18.26 (5.79–57.61)). *HLA-B*∗*15:21* was significantly associated with carbamazepine-induced SJS/TEN appearing in 12.5% (2/16) of cases as compared to 1.48% (4/271) and 0.43% (2/470) in carbamazepine-tolerant controls and general Thai subjects, respectively.

As shown in Table 5, the *HLA-B*∗*58:01* allele was detected as significant in the carbamazepine-induced DRESS group when compared with the carbamazepine-tolerant control group (*P* = 0.032; OR (95% CI) = 7.55 (1.20–47.58)). The *HLA-B*∗*58:01* allele was present in 40.00% (2/5) of the DRESS patients, but in only 8.12% (22/271) of the carbamazepine-tolerant controls and 12.13% (57/470) of the general population.

## 4. Discussion

*HLA-B* alleles are reported to be associated with hypersensitivity reactions during the clinical usage of carbamazepine [25]. Pharmacogenetic screening of *HLA-B* alleles before initiating carbamazepine therapy can prevent the risk of severe and life-threatening cutaneous adverse drug reactions. This study recruited patients with carbamazepine-induced hypersensitivity reactions, such as MPE, DRESS, and SJS/TEN and carbamazepine-tolerant patients from Thailand. We found the association between *HLA-B* alleles (*B*∗*15:02* and *B*∗*58:01*) and carbamazepine-induced MPE. Further, the *HLA-B*∗*15:02* and *HLA-B*∗*15:21* alleles were strongly associated with carbamazepine-induced SJS/TEN, and carbamazepine-induced DRESS had significant association with *HLA-B*∗*58:01* allele.

The evidence of association of different types of carbamazepine-induced cADRs was shown by Hung et al. in Han Chinese patients [3]. In their study, the *HLA-A*∗*31:01* allele was associated with MPE (*P*_*c*_ = 2.2 × 10^−3^; OR (95% CI) = 17.5 (4.6–66.5)) and *HLA-B*∗*15:02* was the susceptible allele for SJS/TEN (*P*_*c*_ = 1.6 × 10^−41^; OR (95% CI) = 1357 (193.4–8838.3)). Few studies have been conducted in the Thai population regarding the involvement of *HLA* alleles in carbamazepine-induced cADRs. The *HLA-A*∗*31:01* allele has been mainly associated with carbamazepine-induced DRESS and MPE in Han Chinese population, Japanese, and European populations [17, 19, 26]. Our study did not perform *HLA-A* typing, and we might have missed the potential association between the *HLA-A*∗*31:01* allele and carbamazepine-induced hypersensitivity reactions.

In 2008, Locharernkul et al. first identified that the *HLA-B*∗*15:02* allele was strongly associated with carbamazepine-induced SJS (*P* = 0.0005) in the Thai population [27]. A consistent association of the cases of carbamazepine-induced SJS/TEN were reported among the carriers of the *HLA-B*^∗^*15:02* allele in this Thai population [20, 28]. Our findings justify the strongest association of the *HLA-B*∗*15:02* allele in the prediction of carbamazepine-induced SJS/TEN. In our study, we observed that the *HLA-B*∗*15:02* allele was not specific for carbamazepine-induced SJS/TEN only, but it was also significantly associated with carbamazepine-induced MPE. However, a previous study by Hung et al. reported the phenotype-specific *HLA* association of carbamazepine-induced cADRs [3]. This discrepancy might be due to the different study populations. We observed the first evidence of a significant association of the *HLA-B*∗*15:21* allele with carbamazepine-induced SJS/TEN in Thai subjects. *HLA-B*^∗^*15:21* allele belongs to the HLA-B75 family, which consists of the *HLA-B*∗*15:02* allele as well [29]. Jaruthamsophon et al. reported that *HLA-B*∗*15:21* was associated with carbamazepine-induced SJS in different populations and that a patient without the *HLA-B*∗*15:02* allele may be at a risk of carbamazepine-induced SJS due to the presence of the *HLA-B*∗*15:21* allele, another HLA-B75 serotype marker [21]. We can conclude that the presence of alternative forms of *HLA* alleles belonging to the same subfamilies of serotypes might contribute to the susceptibility to cADRs. These observations imply that members of the HLA-B75 serotype encode proteins sharing a similar conformation for carbamazepine binding and presentation and trigger the immune response of SJS caused by carbamazepine [19].

In our study, we also found the association of the *HLA-B*∗*58:01* allele with carbamazepine-induced MPE and DRESS. In contrast to our finding, Cheung et al. noted in Han Chinese that the presence of the *HLA-B*∗*58:01* allele appears to be protective against the development of carbamazepine-induced SJS/TEN [30]. A meta-analysis investigating the association of *HLA-B* alleles and carbamazepine-induced SJS/TEN also found that the *HLA-B*∗*58:01* allele was a protective marker among Asian populations [31]. From these observations, we can conclude that genetic susceptibility to carbamazepine-induced cADRs is phenotype-specific. The *HLA-B*∗*58:01* allele is mainly associated with allopurinol-induced MPE, DRESS, and SJS/TEN in the Thai population [22, 32]. There are structural dissimilarities between carbamazepine and allopurinol; therefore, the details of the mechanism, including how exactly the *HLA-B*∗*58:01* allele interacts with each drug and exhibits the immune response, should be explored in future studies. Genetic screening of the *HLA-B*∗*15:02* allele in isolation will fail to prevent carbamazepine-induced MPE/DRESS. The association of the *HLA-B*∗*58:01* allele with carbamazepine-induced MPE and DRESS indicates the role of multiple *HLA-B* alleles, and the genetic testing of these alleles will improve the prevention of carbamazepine-induced cADRs. The *P* value for the association of the *HLA-B*∗*58:01* allele with carbamazepine-induced DRESS is just below the margin of significance (*P* = 0.032). This finding must be considered preliminary and further studies are required to confirm this association of the *HLA-B*∗*58:01* allele with carbamazepine-induced DRESS.

**Figure 1:**
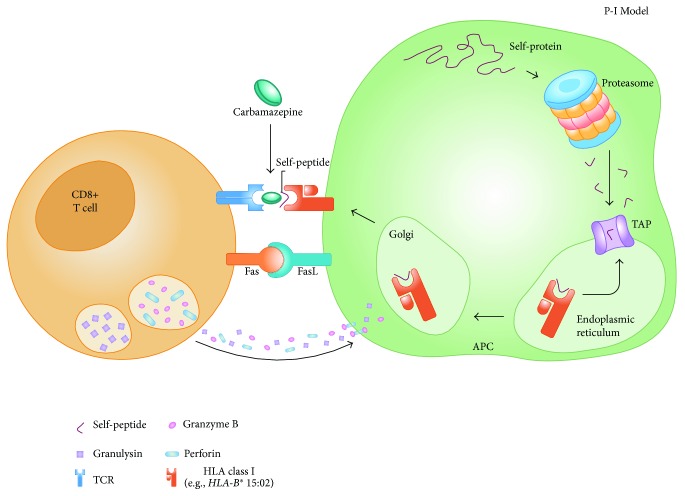
The “pharmacological interaction with immune receptors (p–i)” model of immune activation during carbamazepine-induced hypersensitivity reactions.

The pathogenesis of these carbamazepine-induced hypersensitivity reactions needs further research, due to the role of genetic and host factors in carbamazepine-induced cADRs. The role of carbamazepine-specific T cells and its T cell receptors (TCRs) in the pathogenesis of carbamazepine-induced cADRs must be documented to evaluate the mechanism of carbamazepine-induced cADRs [33]. As illustrated in Figure 1, the “pharmacological interaction with immune receptors (p–i)” concept is a useful model to explain how carbamazepine triggers an immune-mediated hypersensitivity reactions [10].

Our study has provided substantial evidence of the development of MPE, SJS/TEN, and DRESS among carbamazepine-treated patients with *HLA* risk alleles. Screening of the risk alleles before carbamazepine use in the Thai population will significantly reduce cADRs with the exclusion of high-risk patients. We did not carry out an analysis of the involvement of *HLA-A* and *HLA-C* alleles in carbamazepine-induced hypersensitivity reactions, so this might limit the scope of the application of our findings in clinical settings. Therefore, further studies should include association analysis of *HLA-A* and *HLA-C* variants with cADRs in Thai population. The adjusted significance level after Bonferroni's correction is 0.003 with 17 *HLA-B* alleles tested. Only the *HLA-B*∗*15:02* allele remained significant with *P* < 0.003 after Bonferroni adjustment. Because, the smallest *P* value in Tables 2–5 is >0.003, no other alleles are deemed significant after Bonferroni adjustment.

## 5. Conclusions

We found a strong association between the *HLA-B*∗*15:02* allele and carbamazepine-induced SJS/TEN and MPE in Thai patients. We also reported an association of the *HLA-B*∗*15:21* allele with carbamazepine-induced SJS/TEN providing a new perspective of the pharmacogenetic linkage. In addition, the *HLA-B*∗*58:01* allele was also found to be a significant predictor of carbamazepine-induced MPE and DRESS in Thai patients. These findings may need to be confirmed before clinical interpretation and usage with the inclusion of larger sample sizes in further studies. Testing multiple related *HLA* alleles will aid in more reliable evaluation of the risks for developing SJS/TEN and MPE in patients prior to taking carbamazepine.

## Figures and Tables

**Table 1:** Clinical characteristic of patients with carbamazepine-induced cutaneous adverse drug reactions and carbamazepine-tolerant controls.

Demographic data	Cases (*n* = 38)	Tolerant controls (*n* = 271)	*P* value
Gender (*n*/%)			0.145
Male	24/63.15	137/50.6	
Female	14/33.84	134/49.4	
Age (mean/range)	44/24–64	32/10–54	0.010
Indication (*n*/%)			
Epilepsy	29/75.31	108/39.85	2.26 × 10^−5^
Neuropathic pain	2/5.26	23/8.5	0.752
Trigeminal neuralgia	5/13.2	62/22.88	0.173
Bipolar disorder	1/2.6	10/3.7	1.000
Paroxysmal kinesigenic and nonkinesigenic dyskinesia	1/2.6	7/2.6	1.000
Autism	—	35/12.9	0.012
Schizophrenia	—	18/6.6	0.143
Others	—	8/3.0	0.602
Dose of carbamazepine; mg/day (mean ± SD)	325 ± 75	418 ± 19	0.397
Onset of cADRs; days (mean ± SD)	16 ± 7	—	—
cADRs (*n*/%)			
MPE	17/45	—	—
SJS/TEN	16/42	—	—
DRESS	5/13	—	—

cADRs: cutaneous adverse drug reactions; SJS/TEN: Stevens-Johnson syndrome/toxic epidermal necrolysis; DRESS: drug reaction with eosinophilia and systemic symptoms; MPE: maculopapular exanthema.

**Table 2:** Association of *HLA-B* alleles with carbamazepine-induced cADRs.

*HLA-B* alleles	Carbamazepine-induced cADRs (*n* = 38)	Controls (*n* = 271)	Thai population (*n* = 470)	Carbamazepine-induced cADRs cases versus tolerant controls		Carbamazepine-induced cADRs cases versus Thai population	
				OR (95% CI)	*P* value	OR (95% CI)	*P* value
*B*∗*07:05*	3 (7.89%)	12 (4.43%)	24 (5.11%)	1.85 (0.50–6.88)	0.357	1.59 (0.46–5.55)	0.4649
*B*∗*13:01*	1 (2.63%)	37 (13.65%)	54 (11.49%)	0.17 (0.02–1.28)	0.063	0.21 (0.03–1.55)	0.106
*B*∗*13:02*	1 (2.63%)	6 (2.21%)	20 (4.26%)	1.19 (0.14–10.19)	1.000	0.61 (0.08–4.66)	1.000
*B*∗*15:01*	1 (2.63%)	10 (3.69%)	5 (1.06%)	0.71 (0.09–5.67)	1.000	2.51 (0.29–22.08)	0.374
*B*∗*15:02*	17 (44.74%)	11 (4.06%)	71 (15.11%)	19.13 (7.94–46.09)	7.35 × 10^−12^^∗^	4.55 (2.29–9.05)	3.44 × 10^−6^^∗^
*B*∗*15:21*	2 (5.26%)	4 (1.48%)	2 (0.43%)	3.71 (0.66–20.97)	0.161	13.00 (1.78–95.01)	0.030^∗^
*B*∗*18:01*	4 (10.53%)	29 (10.70%)	36 (7.66%)	0.98 (0.33–2.97)	0.974	1.42 (0.48–3.22)	0.529
*B*∗*18:15*	2 (5.26%)	0 (0.00%)	0 (0.00%)	15.06 (1.33–170.25)	0.041^∗^	26.11 (2.31–294.90)	0.016^∗^
*B*∗*27:04*	2 (5.26%)	12 (4.43%)	19 (4.04%)	1.20 (0.26–5.58)	0.685	1.32 (0.30–5.89)	0.665
*B*∗*27:06*	1 (2.63%)	8 (2.95%)	12 (2.55%)	0.89 (0.11–7.31)	1.000	1.03 (0.13–8.15)	1.000
*B*∗*40:01*	5 (13.16%)	41 (15.13%)	58 (12.34%)	0.85 (0.31–2.31)	0.749	1.08 (0.40–2.87)	0.883
*B*∗*44:03*	3 (7.89%)	20 (7.38%)	42 (8.94%)	1.08 (0.30–3.81)	0.910	0.47 (0.14–1.59)	0.223
*B*∗*46:01*	8 (21.05%)	64 (23.62%)	122 (25.96%)	0.86 (0.38–1.98)	0.718	0.77 (0.34–1.72)	0.524
*B*∗*51:01*	5 (13.16%)	21 (7.75%)	40 (8.51%)	1.80 (0.64–5.11)	0.267	1.63 (0.60–4.41)	0.337
*B*∗*56:04*	1 (2.63%)	1 (0.37%)	12 (2.55%)	7.30 (0.45–119.17)	0.231	1.03 (0.13–8.15)	1.000
*B*∗*57:01*	1 (2.63%)	9 (3.32%)	11 (2.34%)	0.79 (0.10–6.39)	1.000	1.13 (0.14–8.98)	0.611
*B*∗*58:01*	8 (21.05%)	22 (8.12%)	57 (12.13%)	3.02 (1.24–7.38)	0.015^∗^	1.93 (0.85–4.42)	0.119

cADRs: cutaneous adverse drug reactions; OR: odds ratio; 95% CI: confidence interval 95%. ^∗^*P* value less than 0.05.

**Table 3:** Association of *HLA-B* alleles with carbamazepine-induced MPE.

*HLA-B* alleles	Carbamazepine-induced MPE (*n* = 17)	Controls (*n* = 271)	Thai population (*n* = 470)	Carbamazepine-induced MPE cases versus tolerant controls		Carbamazepine-induced MPE cases versus Thai population	
				OR (95% CI)	*P* value	OR (95% CI)	*P* value
*B*∗*07:05*	1 (5.88%)	12 (4.43%)	24 (5.11%)	1.44 (0.18–11.81)	0.533	1.234 (0.16–9.78)	0.576
*B*∗*13:02*	1 (5.88%)	6 (2.21%)	20 (4.26%)	2.94 (0.33–26.05)	0.334	1.50 (0.19–11.93)	0.512
*B*∗*15:02*	4 (23.52%)	11 (4.06%)	71 (15.11%)	7.27 (2.04–25.97)	0.002^∗^	2.30 (0.36–4.67)	0.721
*B*∗*18:01*	1 (5.88%)	29 (10.70%)	36 (7.66%)	0.19 (0.03–1.40)	0.098	0.80 (0.10–6.26)	1.000
*B*∗*18:15*	1 (5.88%)	0 (0.00%)	0 (0.00%)	18.07 (1.08–303.14)	0.108	31.33 (1.87–525.19)	0.065
*B*∗*27:04*	1 (5.88%)	12 (4.43%)	19 (4.04%)	1.44 (0.18–11.81)	0.533	1.58 (0.20–12.61)	0.495
*B*∗*40:01*	3 (17.65%)	41 (15.13%)	58 (12.34%)	1.30 (0.35–4.74)	0.720	1.64 (0.45–5.93)	0.438
*B*∗*44:03*	2 (11.77%)	20 (7.38%)	42 (8.94%)	1.72 (0.37–8.11)	0.369	1.46 (0.32–6.62)	0.648
*B*∗*46 :01*	3 (17.65%)	64 (23.62%)	122 (25.96%)	0.69 (0.19–2.49)	0.574	0.66 (0.18–2.35)	0.772
*B*∗*51:01*	3 (17.65%)	21 (7.75%)	40 (8.51%)	2.55 (0.69–9.60)	0.166	2.30 (0.65–8.35)	0.204
*B*∗*57:01*	1 (5.88%)	9 (3.32%)	11 (2.34%)	1.94 (0.23–16.34)	0.442	2.78 (0.34–22.96)	0.334
*B*∗*58:01*	5 (29.41%)	22 (8.12%)	57 (12.13%)	4.74 (1.53–14.66)	0.007^∗^	3.03 (1.03–8.88)	0.045^∗^

MPE: maculopapular exanthema; OR: odds ratio; 95% CI: confidence interval 95%. ^∗^*P* value less than 0.05.

**Table 4:** Association of *HLA-B* alleles with carbamazepine-induced SJS/TEN.

*HLA-B* alleles	Carbamazepine-induced SJS/TEN (*n* = 16)	Controls (*n* = 271)	Thai population (*n* = 470)	Carbamazepine-induced SJS/TEN cases versus tolerant controls		Carbamazepine-induced SJS/TEN cases versus Thai population	
				OR (95% CI)	*P* value	OR (95% CI)	*P* value
*B*∗*07:05*	2 (12.50%)	12 (4.43%)	24 (5.11%)	3.08 (0.63–12.13)	0.165	2.65 (0.57–12.35)	0.213
*B*∗*13:01*	1 (6.25%)	37 (13.65%)	54 (11.49%)	0.40 (0.05–3.07)	0.709	0.48 (0.06–3.70)	0.707
*B*∗*15:01*	1 (6.25%)	10 (3.69%)	5 (1.06%)	1.63 (0.20–13.55)	0.494	5.81 (0.64–52.67)	0.193
*B*∗*15:02*	12 (75.00%)	11 (4.06%)	71 (15.11%)	70.91 (19.67–255.65)	4.46 × 10^−13^^∗^	18.26 (5.79–57.61)	6.9 × 10^−8^^∗^
*B*∗*15:21*	2 (12.50%)	4 (1.48%)	2 (0.43%)	9.54 (1.61–56.57)	0.013^∗^	19.14 (2.51–146.09)	0.004^∗^
*B*∗*18:01*	2 (12.50%)	29 (10.70%)	36 (7.66%)	1.19 (0.26–5.51)	0.822	1.72 (0.38–7.88)	0.483
*B*∗*18:15*	1 (6.25%)	0 (0.00%)	0 (0.00%)	16.94 (1.01–183.39)	0.114	29.38 (1.76–490.97)	0.069
*B*∗*44:03*	1 (6.25%)	20 (7.38%)	42 (8.94%)	0.78 (0.10–6.22)	1.000	0.37 (0.05–2.89)	0.484
*B*∗*46:01*	4 (25.00%)	64 (23.62%)	122 (25.96%)	1.08 (0.34–3.46)	0.899	0.96 (0.30–3.03)	0.947
*B*∗*56:04*	1 (6.25%)	1 (0.37%)	12 (2.55%)	16.88 (1.01–282.35)	0.115	2.39 (0.29–19.48)	0.374
*B*∗*58:01*	1 (6.25%)	22 (8.12%)	57 (12.13%)	0.71 (0.09–5.59)	1.000	0.45 (0.06–3.48)	0.707

SJS/TEN: Stevens-Johnson syndrome/toxic epidermal necrolysis; OR: odds ratio; 95% CI: confidence interval 95%. ^∗^*P* value less than 0.05.

**Table 5:** Association of *HLA-B* alleles with carbamazepine-induced DRESS.

*HLA-B* alleles	Carbamazepine-induced DRESS (*n* = 5)	Controls (*n* = 271)	Thai population (*n* = 470)	Carbamazepine-induced DRESS cases versus tolerant controls		Carbamazepine-induced DRESS cases versus Thai population	
				OR (95% CI)	*P* value	OR (95% CI)	*P* value
*B*∗*15:02*	1 (20.00%)	11 (4.06%)	71 (15.11%)	5.91 (0.61–57.36)	0.126	1.41 (0.16–12.75)	0.562
*B*∗*18:01*	1 (20.00%)	29 (10.70%)	36 (7.66%)	2.09 (0.23–19.30)	0.440	3.01 (0.33–27.68)	0.325
*B*∗*27:04*	1 (20.00%)	12 (4.43%)	19 (4.04%)	5.40 (0.56–52.04)	0.216	5.93 (0.63–55.68)	0.194
*B*∗*27:06*	1 (20.00%)	8 (2.95%)	12 (2.55%)	8.22 (0.82–82.09)	0.154	9.54 (0.99–91.90)	0.130
*B*∗*40:01*	2 (40.00%)	41 (15.13%)	58 (12.34%)	3.74 (0.61–23.08)	0.174	4.74 (0.78–28.94)	0.122
*B*∗*51:01*	1 (20.00%)	21 (7.75%)	40 (8.51%)	2.98 (0.32–27.85)	0.339	2.69 (0.29–24.63)	0.382
*B*∗*58:01*	2 (40.00%)	22 (8.12%)	57 (12.13%)	7.55 (1.20–47.58)	0.032^∗^	4.83 (0.79–29.53)	0.088

DRESS: drug reaction with eosinophilia and systemic symptoms; OR: odds ratio; 95% CI: confidence interval 95%. ^∗^*P* value less than 0.05.
